# Diagnostic accuracy for colorectal cancer of a quantitative faecal immunochemical test in symptomatic primary care patients: a study protocol

**DOI:** 10.1186/s41512-022-00129-7

**Published:** 2022-08-18

**Authors:** Anna Lööv, Cecilia Högberg, Mikael Lilja, Elvar Theodorsson, Per Hellström, Alexandra Metsini, Louise Olsson

**Affiliations:** 1 Department of Medical Sciences, Örebro University, Örebro, Sweden; 2 Skebäck Primary Care Centre, Region Örebro län, Örebro, Sweden; 3 Department of Public Health and Clinical Medicine, Unit of Research, Education and Development Östersund, Umeå University, Umeå, Sweden; 4 Department of Biomedical and Clinical Science; Clinical Chemistry, Linköping University, Linköping, Sweden; 5 Department of Medical Sciences, Uppsala University, Uppsala, Sweden; 6 Department of Knowledge Management and Patient Safety Unit, Region Värmland, Karlstad, Sweden; 7 Centre for Assessment of Medical Technology, Örebro University Hospital, Örebro, Sweden

**Keywords:** Colorectal cancer, Quantitative faecal immunochemical test, Primary care, Sensitivity, Diagnostic accuracy study

## Abstract

**Background:**

There is increasing evidence supporting the use of faecal immunochemical tests (FIT) in patients reporting symptoms associated with colorectal cancer (CRC), but most studies until now have focused on selected subjects already referred for investigation. We therefore set out to determine the accuracy and predictive values of FIT in a primary care population.

**Method:**

A prospective, multicentre, single-gated comparative diagnostic study on quantitative FIT in patients aged 40 years and above presenting in primary care with symptoms associated with CRC will be conducted. Patients representing the whole spectrum of severity of such symptoms met with in primary care will be eligible and identified by GPs.

Participants will answer a short form on symptoms during the last month. They will provide two faecal samples from two separate days. Analyses will be performed within 5 days (QuikRead go®, Aidian Oy). The analytical working range is 10–200 μg Hb/g faeces.

Reference test will be linked to the Swedish Colorectal Cancer Registry up to 2 years after inclusion. Accuracy, area under ROC curves, and predictive values will be calculated for one FIT compared to the highest value of two FIT and at cutoff < 10, 10–14.9, 15–19.9 and ≥ 20 μg Hb/g faeces. Subgroup analyses will be conducted for patients with anaemia and those reporting rectal bleeding. A model-based cost-effectiveness analysis based on the clinical accuracy study will be performed.

Based on previous literature, we hypothesized that the sensitivity of the highest value of two FIT at cutoff 10 μg Hb/g faeces will be 95% (95% CI + / − 15%). The prevalence of CRC in the study population was estimated to be 2%, and the rate of non-responders to be 1/6. In all, 3000 patients will be invited at 30 primary care centres.

**Discussion:**

This study will generate important clinical real-life structured data on accuracy and predictive values of FIT in the most critical population for work-up of CRC, i.e. patients presenting with at times ambiguous symptoms in primary care. It will help establish the role of FIT in this large group.

**Trial registration:**

NCT05156307. Registered on 14 December 2021—retrospectively registered.

## Background

Globally, colorectal cancer (CRC) was the third most common type of cancer in 2020 with 1.9 million new cases, and more than 900,000 deaths, second only to lung cancer [1]. Screening programs are operating in many countries, but participation varies widely and most patients are diagnosed via the clinical route [2]. Symptoms associated with CRC are non-specific and predictive values are generally low [3]. Clinical decision-making for referral is often challenging, e.g. for elderly frail patients who are at an increased risk of CRC but also report bowel symptoms more frequently. Colonoscopy for work-up is costly and requires extensive bowel preparation. The demand is ever-increasing and long waiting times are commonplace.

In this situation, faecal immunochemical tests (FIT) have received increasing attention as an adjunct to the clinical management of patients presenting with symptoms associated with CRC. A systematic review and meta-analysis, based on 10 primary studies, reported FIT to be associated with very high negative predictive values (NPVs) that may correctly rule out CRC and indicated that colonoscopy could be avoided in 75–80% of symptomatic patients [4]. Sensitivity at cutoff 10 μg Hb/g faeces of several FIT for CRC (OC-Sensor, HM-JACKarc, and QuikRead go) has been reported in the range of 92–100% [4–6]. FIT alone, or within a prediction model, is found to be more reliable than merely symptom-based criteria for investigation [7]. But, importantly, almost all of the previous studies have been conducted amongst patients already referred for colonoscopy. Consequently, the results do not apply to a low-risk primary care population where most patients present [8].

However, lately a few studies have been published on the use of FIT in primary care from countries where FIT has already been introduced as a part of the workup of lower gastrointestinal symptoms [9–12]. As such, these studies have focused on patients with non-alarm symptoms, who are not to be referred urgently. But it is well known that the severity of any symptom may be interpreted differently amongst GPs [13], and as follows, the included population may differ between clinical settings and local medical traditions etc. There is a lack of prospective diagnostic studies on FIT in primary care populations that are not restricted to certain symptoms or groups. The crucial question on whether FIT is to be used as a rule-in or rule-out test also remains unresolved [12].

Rectal bleeding is generally considered an alarming symptom of CRC, but it could be very difficult to assess the severity of this symptom from a patient’s history. It is therefore interesting that at least two recent studies found that FIT may identify patients at an increased risk for CRC, even amongst those reporting rectal bleeding [6, 14]. Given the commonness of rectal bleeding, this is an important reason for further investigation of the performance of FIT.

Anaemia is another common first symptom of CRC, and in a screening context, it is associated with a lower sensitivity of FIT [15]. For this reason, it is possible that FIT should not be used in symptomatic patients with anaemia, in order to avoid false-negative outcomes. This is a key aspect of a potential use of FIT amongst symptomatic patients as approximately some 40% of CRC patients overall are expected to be anaemic at the time of diagnosis [16, 17]. Thorough knowledge on the accuracy of FIT, specifically in patients with either anaemia or amongst those who report rectal bleeding, is a decisive aspect on the potential use of FIT in symptomatic patients, in particular for symptomatic patients presenting in primary care.

The use of FIT has likely been found to be a cost-effective strategy for triaging patients who present with lower abdominal symptoms to colonoscopy [5], but again, this analysis was based on studies carried out on patients already referred for colonoscopy, and only one study evaluating FIT in primary care. Further diagnostic accuracy studies based on symptomatic primary care populations are therefore warranted in order to enable updated health economic analyses.

## Methods

### Aim

The aim of this study is to determine the accuracy and predictive values for colorectal cancer of a quantitative FIT in patients presenting in primary care with symptoms associated with colorectal cancer.

### Design

This is a prospective, multicentre, single-gated comparative diagnostic accuracy study of a quantitative FIT in primary care, Sweden, and will be carried out according to the STARD guidelines [18]. Reference test will be linked to a cancer registry at the latest 2 years after the FIT analysis.

### Study setting and participants

This will be a nationwide study, and patients will be invited at primary health care centres located in sparsely populated northern parts, as well as in larger city centres in central Sweden. The catchment population of each primary care centre will vary. The majority of them will be part of public healthcare, but some will be privately managed centres.

Patients from the age of 40 years and above who present in primary care with symptoms possibly associated with CRC are eligible. They must understand Swedish well enough to comply with the study instructions. Any comorbidity, previous history or medication is no hindrance for participation. Anti-coagulation or NSAIDs do not affect the outcome of FIT and patients receiving such treatment can be included [19]. Asymptomatic patients referred for bowel investigation for other reasons (e.g. heredity) are not eligible.

### Participant identification and consent

Participants will be identified by physicians at the participating centres while listening to the history and symptoms reported by patients. Symptoms for study purposes will not be pre-specified, and it will be up to each GP to decide whether the symptoms reported by patients could be associated with CRC, to resemble the real conditions in primary care. The only restriction will be that CRC at some point during the consultation must be considered one of many possible differential diagnoses.

Physicians at participating centres will be encouraged to invite patients from the whole spectrum of severity of symptoms. There will be no specific claims related to the severity of symptoms but a bottom-line is that they must trigger a medical response of any kind. This involves possible alternatives ranging from expectancy and clinical follow-up only (e.g. discussing bowel habits again), to blood tests only (e.g. merely B-haemoglobin), routine faecal haemoglobin tests, rectoscopy only, or routine or urgent referral for complete bowel examination [20].

There will be no specific requirements on the qualifications of physicians identifying eligible patients for the study. It may involve experienced specialists in primary care as well as junior doctors, as this also resembles the real conditions in primary care.

Physicians will briefly inform and invite eligible patients to participate in the study at the consultation. Patients who are interested in participating in the study will be provided with a “study package” and a nurse assistant will demonstrate the content in detail. Patients will bring the study package back home, and if they finally decide to participate in the study, they will sign an enclosed written consent form and enclose this together with their first faecal sample. Patients will be thus identified and invited consecutively at each primary care centre, but they will not be considered included in the study until a signed written informed consent has arrived in the laboratory. First patients, to test study logistics, were included in May 2021.

### Collection of clinical data

In the study package, patients will find a thorough study information and detailed written and pictorial instructions for the collection of faecal samples. In addition, there are two sample collection tubes, two faecal catchers to simplify the sample collection, two transportation tubes, two padded stamped envelopes and two specific referral forms. Patients will provide faecal samples from two separate days. They will be instructed to provide the samples as soon as possible. Patients will send the sample on the same day as a collection or keep it in the fridge overnight. At a few sites, the patients will drop off the samples at the primary health care centre instead of sending them by ordinary mail. Patients are instructed to fill in the date of collection of each sample on the referral forms.

The study package also holds a short questionnaire on the presence of symptoms during the last 4 weeks. The symptoms asked for are rectal bleeding, passage of rectal mucus, abdominal pain, weight loss, and change of bowel habits (more often/seldom and looser/harder). Finally, there is one question on further investigation or clinical follow-up (alternatives as outlined above). Patients will answer these questions at home and enclose the questionnaire with the first sample.

All materials to be returned are identified with serial study numbers. Before leaving the primary care centre with the study package, a nurse assistant will paste a barcode label with a personal ID number on both referral forms. The nurse assistant will also log the date, serial number, age and sex of all patients receiving a study package. The collected data, from all participating centres, will constitute the basic study population and compliance will be calculated using this data as the denominator. The nurse assistant will also make sure there is a recent analysis of B-Hb, within 2 weeks at the most, for each potential study participant.

### FIT analysis

Incoming samples will be analyzed on a daily basis, except for weekends, at three larger laboratories. One is a university clinical research laboratory; and the other two are hospital laboratories accredited according to ISO 17025 and ISO 15189, respectively. Samples will be analyzed within 5 days and kept at room temperature since the collection. The analysis will be performed using the QuikRead go® instrument (Aidian Oy) according to a Standard operating protocol (SOP) from the manufacturer. Laboratory staff will receive specific training for this purpose. A very limited number of laboratory technicians at each laboratory will be involved. Calibrations of the instrument will be carried out every week using QuikRead FOB Positive Controls.

QuikRead go® is a point-of-care FIT system that demonstrated accurate analytical performance in a recent evaluation [21]. The analytical working range of QuikRead go® is 10–200 μg Hb/g faeces (50–1000 ng/ml). Concentrations above this are displayed as > 200 μg/g, and concentrations below are displayed as < 10 μg Hb/g faeces. Accuracy will be calculated for one and two FIT and at cutoff < 10, 10–14.9, 15–19.9 and ≥ 20 μg Hb/g faeces, respectively.

The outcome of the study FIT will not be reported to participating centres. If requested for clinical purposes, physicians will have to order any FIT according to local routines.

### Reference test

The Swedish Colorectal Cancer Registry (SCRCR) is a nationwide registry for CRC with, in practice, complete coverage [22]. Benefitting from the use of personal ID numbers, study participants will be linked to this register at the latest 2 years after the date of the FIT analysis and this will serve as a reference test. In this way, we expect all patients with CRC (ICD-10 C 18–20) will be identified. Date of diagnosis of CRC will be retrieved from SCRCR, and the time period from the date of faecal sample collection until diagnosis will be calculated. Subsite (caecum, ascendens, transversum, descendens, sigmoid and rectum) and TNM stage will be requested. Patients with no CRC registered in the SCRCR within 2 years after FIT analysis will be categorized as “no CRC”. The linkage to SCRCR will also involve data on vital status (including date of death).

A new unknown proportion of patients included in the study will be referred for colonoscopy as part of the clinical workup of their symptoms. For these patients, data from colonoscopy reports will be retrieved to give an account of diagnoses with FIT concentrations below the various chosen thresholds and also to inform the health economic evaluation. Date of investigation, completeness, quality of bowel cleansing, and findings will be extracted. This will include both macroscopic and microscopic findings and be categorized as CRC, advanced adenoma, non-advanced adenoma, inflammatory bowel disease, diverticulosis/diverticulitis, angiodysplasia, and no abnormalities.

### Sample size

Sensitivity for CRC is the most crucial aspect of FIT for potential use in symptomatic patients and we focused our study on the quest for sensitivity as high as possible. Two faecal samples for FIT may be associated with a higher sensitivity [23], and the only previous study available on QuikRead go® reported a sensitivity of 92% for one sample and 100% for the highest value of two analyses [6]. We therefore hypothesized that using maximum value/2 FIT, at cutoff ≥ 10 μg Hb/g faeces, sensitivity is 95%. We also set the lowest acceptable boundary of the 95% confidence interval at 80%. Based on *α* = 0.05, power 80% and the hypothesis of 95% sensitivity (95% CI 80–100) it was estimated that 48 patients with CRC and 2352 without CRC must be included. We also calculated the sample size according to principles provided in a recent paper [24]. In this way, it was estimated that, based on the hypothesis of sensitivity of 95% (95% CI 80–100), at least 50 patients with CRC would be needed. We reasoned false positive are far less harmful than false negatives in this context and accepted a specificity of 70% (95% CI 45–95).

The prevalence of CRC amongst patients with only low-risk symptoms in a study on FIT in primary care in Denmark was 1.5% [9]. In the present study, there will be no restrictions based on the nature or severity of symptoms, and we estimate the prevalence of CRC will be 2% in the eligible population [25]. In other words, 2500 patients, including 50 with CRC, must be included. In the previous study on QuikRead go® [6], the drop-out rate was 22% but the situation will be more advantageous for the present study as patients will receive oral information, practical instructions and all equipment needed to participate at once at their visit to the primary care centre. In all, we estimate the proportion of individuals who will accept a study package but who will not provide any faecal sample to be at most 1/6 (17%; *n* ~ 500). In summary, 3000 patients must accept receiving a study package and finally make up their minds on study participation.

### Statistical analysis

A few subanalyses are planned from the outset. Patients will be categorized according to WHO definition and Hb concentration < 120 g/L for women and < 130 g/L for men will be considered as anaemia [26]. We will estimate accuracy and predictive values for patients with anaemia vs normal haemoglobin and expect the sensitivity of FIT for CRC to be lower in patients with anaemia. We will also estimate accuracy and predictive values for patients reporting rectal bleeding vs those who do not report rectal bleeding. We expect sensitivity to be higher amongst patients reporting rectal bleeding.

### Health economic evaluation

This study is going to assess the cost-effectiveness of the different diagnostic strategies. The approach of the analysis will be a model-based cost-effectiveness analysis of the present clinical accuracy study. The following strategies will be compared.:

- Triage using FIT (QuikRead go® Aidian Oy), at a threshold of 10 μg Hb/g faeces
- Triage standard (referral straight to colonoscopy)

A diagnostic model-decision tree will be developed in order to represent the different diagnostic pathways for CRC. A measure of effectiveness will be regarded as the detection of true cases of CRC based on the results from the accuracy analysis. The cost per case detected for each diagnostic strategy and the incremental cost-effectiveness ratio will be estimated (ICER) [27]. In this case, the ICER will be the incremental cost per incremental number of true cases of CRC detected and will be calculated as follows:

$$\mathrm{ICER}\:=\:\mathrm{CtrFIT}\:-\:\mathrm{CtrSt}/\mathrm{NCtrFIT}\:-\:\mathrm{NCtrSt}$$

where CtrFIT = cost per case detected associated with triage using FIT, CtrSt = cost per case deteted associated with triage standard, NCtrFIT = number of CRC detected with triage using FIT, and NCtrSt = number of CRC detected with standard triage.

The analysis will adopt a healthcare perspective with time interval from the beginning of the screening (delivery of screening strategy) until the result of the test has been obtained. All resource use and relevant costs that will occur in this time frame will be estimated.

Data on resource use will reflect “real” life practice for the two strategies. Unit costs related to the colonoscopy will be obtained from the Cost-Per-Patient database [28]. Unit cost data of FIT, such as acquisition cost, materials/reagents costs, and maintenance costs, will be obtained from the manufacturer. Use of other healthcare resources, like consultations, various contacts with healthcare professionals (such as information/help from nurses-assistants, possible telephone contacts or on-site visits) will be estimated based on input from the accuracy study and healthcare professionals’ insights. Number and type of possible adverse events per triage strategy will be obtained from the accuracy study. Costs related to managing possible adverse events (for example with colonoscopy, bleeding, polypectomy, perforation) will be estimated based on data obtained from the Cost-Per-Patient database using the relevant procedure codes if possible (for example JFA15 for polypectomy in the colon) or otherwise through consultation with experts on the resources needed. Possible indirect costs, like administration, management, and data quality assessment will be estimated after consultation with involved health care professionals and activity times will be obtained through consultation with personnel performing each task. All labour time will be valued using the average monthly salary for each profession involved obtained from SCB Statistics Sweden.

The base case value scenario for each cost item will be estimated based on the resource utilization multiplied by unit cost and min–max values will be reported if this is possible from the input data (for example the Cost-Per-Patient database reports information on min–max values). Total cost per screening strategy, cost per case detected and ICERs will be estimated, and min–max values will be reported if possible. All costs will be reported in 2022 € euros (SEK will be converted to Euros with the exchange rate at the time of the analysis).

The robustness of the results will be explored with sensitivity analysis [29], such as one-way deterministic sensitivity analysis which investigates how the results (base case scenario) are affected by the change of the value of one parameter individually (e.g. change of the sensitivity level, use of min–max values of unit costs or resource use). Additionally, ICERs will be estimated by subgroups based on input from the accuracy analysis, such as patients reporting rectal bleeding and patients with anaemia och type of findings such as CRC stage.

The analysis will also include a budget impact analysis (BIA) associated with the implementation of triage using FIT in the primary health care setting in Sweden [30]. The total health care cost will be estimated for the different screening strategies based on the estimation of the eligible population in Sweden for screening for CRC in the primary care setting and the cost per patient for each screening strategy (base case scenario). The influence on the health care budget will be explored based on different rates of substitution of the current screening praxis with the FIT.

## Discussion

There is increasing evidence in support of FIT as a triage test for selection for further investigation in patients reporting symptoms possibly associated with colorectal cancer in primary care. In the UK, such a pathway (NICE guidance DG30) has already been introduced to guide referral for patients in primary care who report unexplained symptoms but do not fulfil the criteria for a suspected cancer pathway [31].

An accurate triage test for workup of symptoms that could be associated with CRC is obviously beneficial to avoid an ample part of diagnostic colonoscopies, but it presumes there is solid proof on sensitivity. Any sensitivity lower than for colonoscopy itself holds a risk of delaying CRC diagnoses in a small, but a symptomatic number of patients presenting in healthcare with their concerns. It is therefore crucial to provide more data based on symptomatic primary care patients on the consequences of using FIT as a rule-out test. A recent study from the UK evaluated the use of FIT according to current guidelines on the management of low-risk symptoms and reported a sensitivity of merely 84% (95% 71–93) [11]. It is lower than in previous studies and may indicate that FIT is better used as a risk stratification tool to prioritize amongst patients referred for colonoscopy.

However, we suggest that one way forward is to identify subgroups of patients for whom the use of FIT is not beneficial and who should be referred for colonoscopy firsthand. Anaemic patients may constitute such a group. From screening, it is known that right-sided advanced neoplasia is associated with a lower sensitivity for a faecal occult blood test [15].

A recent study on FIT-negative CRC, also based on current UK guidelines, reported 6/7 had caecal cancer and 5 of them had anaemia [32]. And the reverse, haemoglobin levels above the limit for anaemia indicated a low risk of CRC in FIT-negative patients in a Swedish study [24]. We conclude it is of vital interest to further investigate the association between the outcome of FIT and B-Hb.

One advantage of evaluating a quantitative FIT is the ability to vary the cut-off of faecal haemoglobin concentration in relation to the purpose of the test. In this way, we will be able to compare the outcome of this study with many previous studies on qualitative FIT, ie mainly using a cutoff at 10 μg Hb/g faeces. But we will also be able to calculate the consequences of adopting a much higher cut-off and evaluating the effects of using the test for prioritization purposes for colonoscopy.

The number of patients presenting in Swedish primary care with symptoms possibly associated with CRC is not known. In general, the threshold for referral for colonoscopy is low but the use of colonoscopies varies manifold between different parts of the country according to the Swedish National Board of Health and Welfare’s (Socialstyrelsen) statistics database [33]. The tendency to present with symptoms in primary care varies between different groups, gender is one but many other factors are plausible. The present study will involve not only patients who are referred for further investigation according to guidelines but, importantly, also patients who are carefully assessed by GPs who did not find the indication for complete bowel examination strong enough. It follows that the size of this particular group of patients is not well known and that it varies with many factors. Nevertheless, we understand that without this difficult aspect of clinical work, the demand for colonoscopy would be even higher. Better knowledge on the performance of FIT in this group is warranted.

The reference test for all participants will be linked to the SCRCR. Calculations on accuracy will be based on dichotomization of CRC vs no CRC, and information on other possible lesions causing a positive FIT, will not be available. This is a drawback but the best option available is to identify study participants with a CRC diagnosis. A second reference test, colonoscopy, will be used but inevitably it will only be available for more high-risk patients.

The incidence of CRC is increasing amongst younger age groups [34], and young age can no longer be taken as a protective factor against CRC as previously. This adds to the importance of more accurate preselection tests prior to colonoscopy, beyond mere symptoms alone. The majority of patients would also prefer a FIT over colonoscopy given that both tests have the same sensitivity [35].

The main challenge of this study is in the numbers needed. Some 30 primary care centres are already involved but given the pressure on primary care services, it is still a challenging undertaking. This is accentuated by the fact that neither patients nor GPs will benefit from participation as the outcome of the study FIT will remain within the study, and if FIT is requested for clinical reasons, it must be provided separately. However, the study has been well received as the potential benefit of FIT amongst patients in primary care is imaginable to most stakeholders.

## Data Availability

The dataset generated by the current study will not be publicly available immediately, as possible follow-up studies may be conducted. Once they are completed, the question will be resettled.

## Abbreviations

BIA: Budget impact estimation
CEA: Cost-effectiveness analysis
CEAC: Cost-effectiveness acceptability curves
CRC: Colorectal cancer
FIT: Faecal immunochemical test
GP: General practitioner
ICER: Incremental cost-effectiveness ratio
NPV: Negative predictive value
PSA: Probabilistic sensitivity analysis
SCRCR: Swedish Colorectal Cancer Registry
WTP: Willingness-to-pay

## References

[1] World Health Organization. https://gco.iarc.fr/today/data/factsheets/cancers/39-All-cancers-fact-sheet.pdf. Accessed 8 Dec 2021.

[2] Wools A, Dapper EA, de Leeuw JR (2016). Colorectal cancer screening participation: a systematic review. Eur J Public Health.

[3] Adelstein BA, Macaskill P, Chan SF, Katelaris PH, Irwig L (2011). Most bowel cancer symptoms do not indicate colorectal cancer and polyps: a systematic review. BMC Gastroenterol.

[4] Westwood M, Lang S, Armstrong N, van Turenhout S, Cubiella J, Stirk L (2017). Faecal immunochemical tests (FIT) can help to rule out colorectal cancer in patients presenting in primary care with lower abdominal symptoms: a systematic review conducted to inform new NICE DG30 diagnostic guidance. BMC Med.

[5] Westwood M, Corro Ramos I, Lang S, Luyendijk M, Zaim R, Stirk L (2017). Faecal immunochemical tests to triage patients with lower abdominal symptoms for suspected colorectal cancer referrals in primary care: a systematic review and cost- effectiveness analysis. Health Technol Assess.

[6] Tsapournas G, Hellström PM, Cao Y, Olsson LI (2020). Diagnostic accuracy of a quantitative faecal immunochemical test vs. symptoms suspected for colorectal cancer in patients referred for colonoscopy. Scand J Gastroenterol..

[7] Herrero JM, Vega P, Salve M, Bujanda L, Cubiella J (2018). Symptom or faecal immunochemical test based referral criteria for colorectal cancer detection in symptomatic patients: a diagnostic tests study. BMC Gastroenterol.

[8] Leeflang MM, Rutjes AW, Reitsma JB, Hooft L, Bossuyt PM (2013). Variation of a test's sensitivity and specificity with disease prevalence. CMAJ.

[9] Juul JS, Hornung N, Andersen B, Laurberg S, Olesen F, Vedsted P (2018). The value of using the faecal immunochemical test in general practice on patients presenting with non- alarm symptoms of colorectal cancer. Br J Cancer.

[10] Nicholson BD, James T, Paddon M (2020). Faecal immunochemical testing for adults with symptoms of colorectal cancer attending English primary care: a retrospective cohort study of 14 487 consecutive test requests. Aliment Pharmacol Ther.

[11] Bailey SER, Abel GA, Atkins A, Byford R, Davies SJ, Mays J (2021). Diagnostic performance of a faecal immunochemical test for patients with low-risk symptoms of colorectal cancer in primary care: an evaluation in the South West of England. Br J Cancer.

[12] Chapman C, Thomas C, Morling J, Tangri A, Oliver S, Simpson JA (2020). Early clinical outcomes of a rapid colorectal cancer diagnosis pathway using faecal immunochemical testing in Nottingham. Colorectal Dis.

[13] Baughan P, Keatings J, O'Neill B (2011). Urgent suspected cancer referrals from general practice: audit of compliance with guidelines and referral outcomes. Br J Gen Pract.

[14] Högberg C, Gunnarsson U, Cronberg O, Thulesius H, Lilja M, Jansson S (2020). Qualitative faecal immunochemical tests (FITs) for diagnosing colorectal cancer in patients with histories of rectal bleeding in primary care: a cohort study. Int J Colorectal Dis.

[15] Haug U, Kuntz KM, Knudsen AB, Hundt S, Brenner H (2011). Sensitivity of immunochemical faecal occult blood testing for detecting left- vs right-sided colorectal neoplasia. Br J Cancer.

[16] Li M, Cao Y, Olsson L (2021). A population-based study on time trends of hemoglobin in primary care comparing prediagnostic colorectal cancer patients vs age- and sex-matched controls. Scand J Gastroenterol.

[17] Väyrynen JP, Tuomisto A, Väyrynen SA, Klintrup K, Karhu T, Mäkelä J (2018). Preoperative anemia in colorectal cancer: relationships with tumor characteristics, systemic inflammation, and survival. Sci Rep..

[18] Cohen JF, Korevaar DA, Altman DG, Bruns DE, Gatsonis CA, Hooft L, Irwig L, Levine D, Reitsma JB, de Vet HC, Bossuyt PM. STARD 2015 guidelines for reporting diagnostic accuracy studies: explanation and elaboration. BMJ Open. 2016;6(11). 10.1136/bmjopen-2016-012799. 10.1136/bmjopen-2016-012799 PMC5128957 28137831

[19] Wilkens J, Thulesius H, Schmidt I, Carlsson C (2016). The 2015 National Cancer Program in Sweden: Introducing standardized care pathways in a decentralized system. Health Policy.

[20] Nieuwenburg SAV, Vuik FER, Kruip MJHA, Kuipers EJ, Spaander MCW (2019). Effect of anticoagulants and NSAIDs on accuracy of faecal immunochemical tests (FITs) in colorectal cancer screening: a systematic review and meta-analysis. Gut.

[21] O'Driscoll S, Carroll M, Maclean W, Piggott C, Jourdan I, Benton SC (2021). Assessment of the analytical performance of point-of-care faecal immunochemical tests for haemoglobin. Ann Clin Biochem.

[22] Moberger P, Sköldberg F, Birgisson H (2018). Evaluation of the Swedish Colorectal Cancer Registry: an overview of completeness, timeliness, comparability and validity. Acta Oncol.

[23] Auge JM, Fraser CG, Rodriguez C, Roset A, Lopez-Ceron M, Grau J (2016). Clinical utility of one versus two faecal immunochemical test samples in the detection of advanced colorectal neoplasia in symptomatic patients. Clin Chem Lab Med.

[24] Korevaar DA, Gopalakrishna G, Cohen JF, Bossuyt PM (2019). Targeted test evaluation: a framework for designing diagnostic accuracy studies with clear study hypotheses. Diagn Progn Res.

[25] Högberg C, Gunnarsson U, Jansson S, Thulesius H, Cronberg O, Lilja M (2020). Diagnosing colorectal cancer in primary care: cohort study in Sweden of qualitative faecal immunochemical tests, haemoglobin levels, and platelet counts. Br J Gen Pract.

[26] World Health Organization. https://apps.who.int/iris/bitstream/handle/10665/85839/WHO_NMH_NHD_MNM_11.1_eng.pdf?sequence=22&isAllowed=y. Accessed 8 Dec 2021.

[27] Drummond MF, Sculpher, M.J., Torrance, G.W., O'Brien, B.J., Stoddartm, G.L. Methods for the Economic Evaluation of Health Care Programmes. 3rd ed: Oxford University Press; 2005.

[28] Cost-per-patient Database. Sveriges Kommuner och Regioner, Stockholm. https://skr.se/skr/halsasjukvard/ekonomiavgifter/kostnadperpatientkpp/kppdatabas.46722.html. Accessed 17 Januari 2022.

[29] Edlin R (2015). Cost Effectiveness Modelling for Health Technology Assessment.

[30] Sullivan SD, Mauskopf JA, Augustovski F, Jaime Caro J, Lee KM, Minchin M, Orlewska E, Penna P, Rodriguez Barrios JM, Shau WY (2014). Budget impact analysis-principles of good practice: report of the ISPOR 2012 Budget Impact Analysis Good Practice II Task Force. Value Health.

[31] National Institute for Health and Care Excellence. https://www.nice.org.uk/guidance/dg30. Accessed 8 Dec 2021.

[32] Cunin L, Khan AA, Ibrahim M, Lango A, Klimovskij M, Harshen R (2021). FIT negative cancers: A right-sided problem? Implications for screening and whether iron deficiency anaemia has a role to play. Surgeon.

[33] Statistics database for operations. National Board of Health and Welfare, Stockholm. https://sdb.socialstyrelsen.se/if_ope/val.aspx. Accessed 20 Dec 2021.

[34] Siegel RL, Torre LA, Soerjomataram I, Hayes RB, Bray F, Weber TK (2019). Global patterns and trends in colorectal cancer incidence in young adults. Gut.

[35] von Wagner C, Verstraete W, Hirst Y, Nicholson BD, Stoffel ST, Laszlo H. Public preferences for using quantitative faecal immunochemical test versus colonoscopy as diagnostic test for colorectal cancer: evidence from an online survey. BJGP Open. 2020;4(1). doi: 10.3399/bjgpopen20X101007. 10.3399/bjgpopen20X101007 PMC7330201 32019773

